# Treatment of *EGFR* mutation–positive non–small cell lung cancer complicated by Trousseau syndrome with gefitinib followed by osimertinib: a case report

**DOI:** 10.18632/oncotarget.25687

**Published:** 2018-06-29

**Authors:** Yoshikane Nonagase, Masayuki Takeda, Kaoru Tanaka, Hidetoshi Hayashi, Tsutomu Iwasa, Kazuhiko Nakagawa

**Affiliations:** ^1^ Department of Medical Oncology, Kindai University Faculty of Medicine, Osaka, Japan

**Keywords:** lung cancer, gefitinib, osimertinib, Trousseau syndrome, EGFR mutation

## Abstract

Malignant tumors can induce a hypercoagulable state known as Trousseau syndrome that increases the risk for venous thromboembolism including disabling cerebral infarction. Anticoagulant therapy without anticancer treatment is not effective for amelioration of this coagulation abnormality. Most patients with lung cancer positive for activating mutations of the epidermal growth factor receptor (EGFR) are sensitive to EGFR tyrosine kinase inhibitors (TKIs), but the efficacy and safety of EGFR-TKIs in such patients with a poor performance status (PS) due to Trousseau syndrome has been unclear. We here describe a patient with *EGFR* mutation–positive lung cancer who developed disabling cerebral infarction due to Trousseau syndrome. Administration of the EGFR-TKI gefitinib and anticoagulant therapy resulted in a partial tumor response and recovery from both the coagulation abnormality and the severe neurological symptoms. After the development of resistance to gefitinib, the EGFR-TKI osimertinib was safely administered until disease progression without recurrence of the coagulation abnormality. This case suggests that gefitinib followed by osimertinib may be a safe and effective treatment option for patients with *EGFR* mutation–positive lung cancer who experience disabling cerebral infarction due to Trousseau syndrome.

## INTRODUCTION

Trousseau syndrome is a state of hypercoagulability associated with certain malignant tumors and can manifest as deep venous thrombosis or cerebral infarction [1]. Given that platinum-based chemotherapy increases the risk of thromboembolic complications, patients with lung cancer and severe cerebral infarction due to Trousseau syndrome are often considered to be ineligible for such treatment [2]. Epidermal growth factor receptor (EGFR) tyrosine kinase inhibitors (TKIs) are now a standard first-line therapy for patients with advanced non–small cell lung cancer (NSCLC) positive for activating *EGFR* mutations, with these drugs also being efficacious and tolerable even in individuals with a poor Eastern Cooperative Oncology Group (ECOG) performance status (PS) associated with disease progression [3]. We now report a case of acute cerebral infarction due to Trousseau syndrome in a patient who was recently diagnosed with lung cancer positive for an activating *EGFR* mutation and who was subsequently treated safely with the EGFR-TKIs gefitinib and osimertinib.

## CASE REPORT

**Figure 1:**
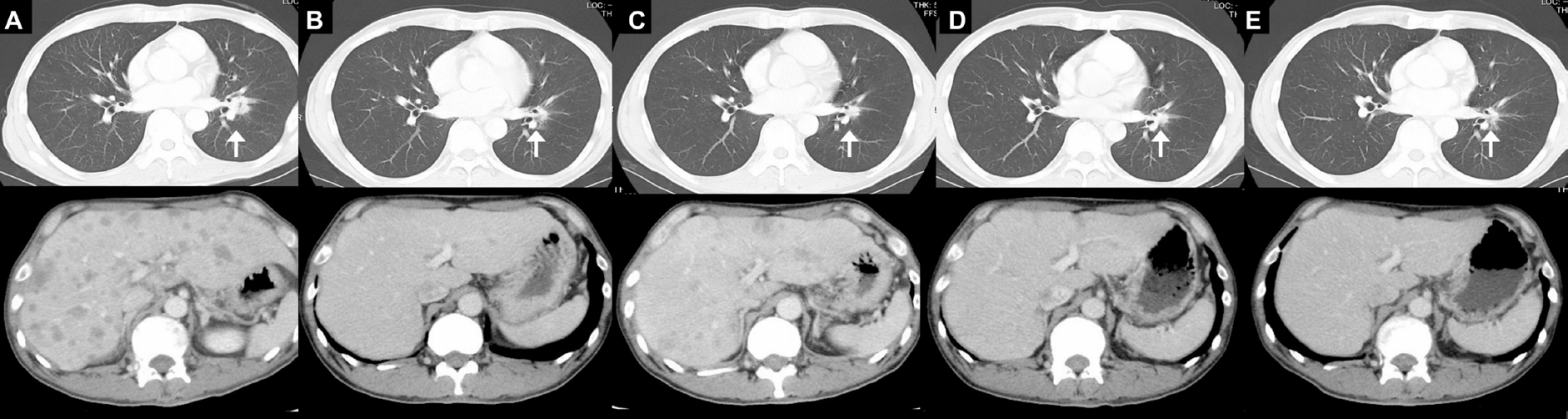
Contrast-enhanced CT scans of the lungs and liver Images were acquired before treatment with gefitinib (**A**), 11 days after the onset of gefitinib treatment (**B**), 7 months after treatment onset, when disease progression was apparent for the liver metastases (**C**), 1 month after the onset of treatment with osimertinib (**D**), and after 3 months of treatment with osimertinib, when leptomeningeal metastasis had occurred (**E**). Arrows indicate primary lung lesion.

A 53-year-old man with no medical history of arrhythmia, diabetes mellitus, coagulation disorder, or stroke consulted our hospital complaining of back pain. Contrast-enhanced computed tomography (CT) showed a 21-mm-diameter nodule in the lower left lung as well as multiple liver and bone metastases (Figure 1A), but no abnormalities in the brain. Percutaneous needle biopsy of the liver led to a diagnosis of adenocarcinoma of the lung (T1bN3M1c, cStage IVb) positive for an exon 19 deletion of the *EGFR* gene.

**Figure 2:**
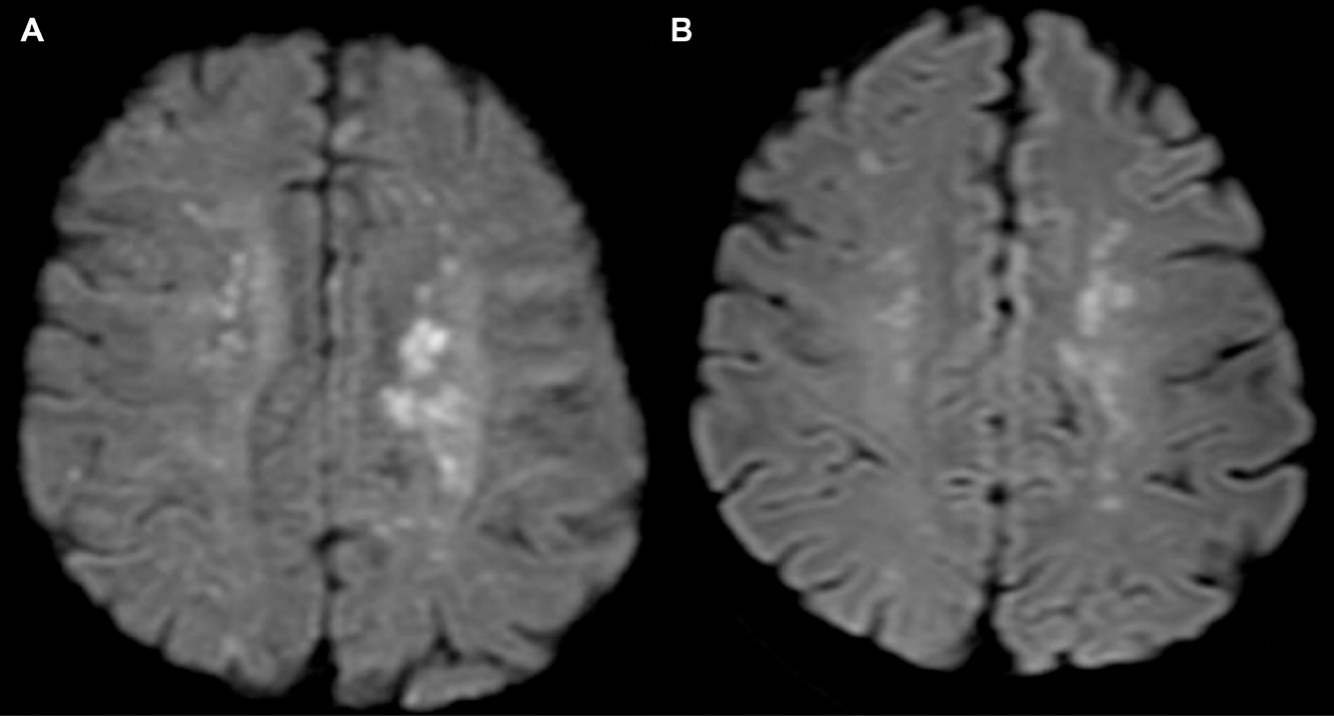
DW-MRI imaging of the brain Images were acquired after acute cerebral infarction (**A**) and 11 days after the onset of treatment with gefitinib together with anticoagulant therapy (**B**).

Before initiation of treatment with gefitinib, the patient was admitted to the hospital because of a disturbance of consciousness and malaise. His ECOG PS was 4. A brain CT scan again showed no abnormalities, whereas laboratory tests revealed a decreased platelet count of 59,000/μl (normal range, 158,000 to 348,000/μl), an increased prothrombin time/international normalized ratio (PT-INR) of 1.35 (normal range, 0.90 to 1.10), and an increased fibrin degradation product level of 174.3 μg/ml (normal range, 0 to 8 μg/ml), suggestive of cancer-associated disseminated intravascular coagulation. Anticoagulant therapy with thrombomodulin alfa (380 U/kg) was initiated. Transthoracic echocardiography revealed no findings of valvular disease or intracardiac thrombus. On his second day in hospital, the patient was started on gefitinib at 250 mg/day, given that this drug has been shown to be safe and effective in *EGFR* mutation–positive NSCLC patients with a poor PS [3]. After 2 days of treatment with gefitinib, the patient presented with right hemiplegia, aphasia, and cognitive dysfunction. Diffusion-weighted magnetic resonance imaging (DW-MRI) revealed multiple acute cerebral infarctions (Figure 2A) and the patient was diagnosed with Trousseau syndrome. He received intravenous unfractionated heparin with a target activated partial thromboplastin time of 40 to 60 s (normal range, 26 to 35 s) for 7 days, and he was treated with warfarin to maintain his PT-INR between 1.5 and 2.5. The hemiplegia, aphasia, and cognitive dysfunction were gradually ameliorated. After 11 days of treatment with gefitinib, laboratory data showed an improvement in the platelet count from 48,000 to 480,000/μl, CT revealed a partial tumor response according to RECIST criteria (Figure 1B), and DW-MRI detected no further cerebral infarction (Figure 2B). After treatment with gefitinib for 7 months, the patient showed disease progression with regard to the liver metastases (Figure 1C) without worsening of his coagulant profile. Rebiopsy of liver metastases to examine status for the T790M resistance mutation of *EGFR* was difficult because of the small lesion size and the continuation of anticoagulant therapy. We were able to detect the T790M mutation by analysis of plasma cell-free tumor DNA with the cobas EGFR Mutation Test (Roche), however, allowing a switch to osimertinib only 5 days after disease progression during gefitinib treatment. Exacerbation of the coagulation abnormality did not recur during treatment with osimertinib. Although shrinkage of the primary lung lesion and liver metastases was maintained during treatment with osimertinib (Figure 1D, 1E), the patient showed leptomeningeal disease progression 3 months after initiation of this therapy. No further chemotherapy was administered because of his poor ECOG PS associated with disease progression, and he died 1 month after discontinuation of osimertinib.

## DISCUSSION

There is no established therapy for patients with *EGFR* mutation–positive NSCLC and Trousseau syndrome who present with severe neurological symptoms. In the present case, consecutive treatment with the EGFR-TKIs gefitinib and osimertinib was safe and effective until disease progression. To our knowledge, this is the first report of such treatment leading to a good response and recovery from neurological symptoms without recurrence of the coagulation abnormality in a patient with *EGFR* mutation–positive NSCLC and Trousseau syndrome.

Anticoagulant therapy alone for cancer-associated thromboembolism is often not effective without concomitant anticancer treatment [4]. Given that malignant cells produce and release procoagulant factors including mucins observed in the present case, amelioration of the coagulation abnormality might require a reduction in tumor volume [5, 6]. Conventional cytotoxic chemotherapy is not routinely administered to patients with a poor ECOG PS linked to disease progression, and platinum-based chemotherapy can increase the risk of thromboembolism [2]. In contrast, EGFR-TKIs can be administered safely even to patients with a poor ECOG PS, and they are associated with a higher tumor response rate and shorter time to response compared with chemotherapy [3, 7]. EGFR-TKIs may thus be a better treatment option than chemotherapy for patients with *EGFR* mutation–positive NSCLC complicated by Trousseau syndrome.

In the present case, exacerbation of the coagulation abnormality did not recur during treatment with gefitinib followed by osimertinib, possibly as a result of the associated marked reduction in tumor volume. The risk for recurrence of venous thromboembolism in cancer patients is thought to be higher for those with more extensive disease [8]. In our case, the tumor volume during gefitinib and osimertinib treatment as well as after subsequent disease progression was much smaller than that before initiation of anticancer therapy. The pronounced reduction in tumor burden achieved with gefitinib and osimertinib might thus have contributed to lowering of the risk for recurrence of venous thromboembolism. We cannot exclude the possibility that the combination of chemotherapy and anticoagulant therapy may have been responsible for the lack of recurrence of thromboembolic events. The recurrence rate for cerebral infarction in patients with cancer was found to be 16% within 6 months, whereas that in patients without cancer receiving anticoagulant therapy was 5.9 to 10.3% within the 1st year [9, 10].

A case of NSCLC associated with Trousseau syndrome was previously found to be controlled with gefitinib followed by erlotinib and then by carboplatin plus pemetrexed [11]. However, the coagulation abnormality recurred several months after initiation of each regimen, presumably because no substantial tumor shrinkage was obtained. Moreover, osimertinib had not been approved at the time of this previous case. For the present case, we were able to switch from gefitinib to osimertinib promptly as a result of the rapid determination of T790M mutation status by liquid biopsy. The results of a recent phase III trial of osimertinib in comparison with gefitinib in previously untreated patients with *EGFR* mutation–positive advanced NSCLC suggest the possibility that osimertinib may be a treatment option for chemonaïve patients with *EGFR*-mutated NSCLC and Trousseau syndrome [12].

In conclusion, gefitinib followed by osimertinib may be a safe and effective treatment option for *EGFR* mutation–positive NSCLC patients with a poor ECOG PS due to Trousseau syndrome.
